# Barriers to adherence in time-restricted eating clinical trials: An early preliminary review

**DOI:** 10.3389/fnut.2022.1075744

**Published:** 2023-01-12

**Authors:** Monica A. O'Neal, Nikko Rigor Gutierrez, Kyla L. Laing, Emily N. C. Manoogian, Satchidananda Panda

**Affiliations:** Regulatory Biology Department, Salk Institute for Biological Studies, La Jolla, CA, United States

**Keywords:** time-restricted eating (TRE), time-restricted feeding (TRF), intermittent fasting (IF), adherence–compliance–persistence, community dwelling adults, dietary intervention, barriers and facilitative factors, behavioral intervention

## Abstract

Time-restricted eating (TRE) has shown potential benefits in optimizing the body's circadian rhythms and improving cardiometabolic health. However, as with all dietary interventions, a participant's ability to adhere to the protocol may be largely influenced by a variety of lifestyle factors. In TRE trials that reported participants' rates of adherence, the percentage of total days with successful adherence to TRE ranged from 47% to 95%. The purpose of this review is to (1) summarize findings of lifestyle factors affecting adherence to TRE clinical trials outside of the lab, and (2) explore a recommended set of behavioral intervention strategies for the application of TRE. A literature search on Pubmed was conducted to identify clinical TRE studies from 1988 to October 5, 2022, that investigated TRE as a dietary intervention. 21 studies included daily self-monitoring of adherence, though only 10 studies reported a combination of family, social, work, and miscellaneous barriers. To maximize participant adherence to TRE and increase the reliability of TRE clinical trials, future studies should monitor adherence, assess potential barriers, and consider incorporating a combination of behavioral intervention strategies in TRE protocols.

## 1. Introduction

The last decade has seen a proliferation of clinical trials investigating the effects of time-restricted eating (TRE) on a myriad of health outcomes. TRE is an eating-fasting schedule in which the eating window is limited to a consistent 6–10 h per day, with some studies including 4–12 h eating windows (1–3). TRE is thought to facilitate nutrient homeostasis *via* the synchronization of metabolic processes to optimal times of each circadian cycle (4), and results have been promising for the improvement of various health outcomes such as weight loss, blood pressure, and glycemic control (5–8). Recent trials have also reported significant increases in insulin sensitivity (1, 9) as well as reductions in fat mass and/or body weight (10–14). Additionally, the benefits of TRE have encompassed improvements in sleep quality and quality of life (15–17). As a novel dietary intervention, TRE is unique in that it demands minimal medical supervision, does not require calorie counting or dietary restrictions, and can be adaptable to individual needs. The methodology across TRE trials has varied, ranging from controlled laboratory settings to implementation in community-dwelling individuals with little to no supervision. This review focuses on community-dwelling individuals, where TRE was incorporated into participants' regular daily lives—which often include family, work, and social obligations.

Despite being a relatively simple dietary intervention, TRE is still a lifestyle change, so the implementation of TRE—particularly in community-dwelling individuals—has posed some challenges. Social events, family obligations, and work commutes are external barriers that commonly influence an individual's ability to maintain a consistent eating window (18). On the other hand, psychological factors (e.g., stress and boredom) can evoke erratic eating patterns induced by stress-eating and compulsive snacking (19). The capacity for individuals to adhere to TRE provides valuable insight into its feasibility as a lifestyle; yet the confluence of external and psychological barriers—and, more importantly, how to mitigate them from a research methodology standpoint—is poorly understood. As participants' adherence to dietary interventions forms the basis of conclusions we can draw from clinical trials, increasing rates of adherence will improve our overall understanding of the impacts of TRE. In light of the challenges associated with adherence to TRE, there is a clear need to (1) explore potential patterns in barriers to adherence across TRE trials, and (2) develop TRE protocols that adapt to the inherent variability of community-dwelling individuals. We have yet to truly understand whether TRE is feasible as a long-term dietary intervention and/or component of a healthy lifestyle.

Data on barriers to TRE adherence and methods of improvement are currently limited. Of the 66 published TRE clinical trials, only 10 reported details on barriers to adherence. This review will summarize findings from these studies on lifestyle factors affecting TRE adherence, summarize participants' methods of overcoming barriers, explore how a set of behavioral intervention strategies may be applied to the TRE protocol, and provide suggestions for improving adherence in future trials.

## 2. Reported barriers to TRE

### 2.1. Socio-environmental factors

A common barrier to TRE adherence across trials was socio-environmental factors (Table 1). External pressures and responsibilities such as family commitments, work schedules, and social occasions can be challenges to maintaining TRE.

**Table 1:** Reported barriers to adherence in TRE trials.

**References**	**Study design**	**Participants (total; completed)**	**Baseline characteristics**	**Age (mean or range)**	**TRE intervention (eating window duration)**	**Duration**	**Barrier categories**	**Frequency of barriers (# of participants)**	**Description of barriers**
Tinsley et al. (12)	RCT	T: *n* = 28; C: *n* = 18	Healthy	22 (SD = 2.4)	4 h (6p−10p)	4 days/week for 8 weeks	Social	Not reported	Social eating opportunities
Antoni et al. (20)	NRXT	T: *n* = 16; C: *n* = 13	Healthy	29–54	Shortened eating window by 3 h (delayed the first meal and advanced last meal by 1.5 h each)	10 weeks	Social	Not reported	Social eating/drinking events
Parr et al. (19)	RXT	T: *n* = 14; C: *n* = 11	Overweight/obese	38.5 (SD = 5)	8 h (meals at 10 am, 1 pm, and 5 pm)	5 days each arm w/10-day washout	Family, work, and social	Work: 11 of 11; social life: 8 of 11; family life: 5 of 11	The main barriers to TRE were work schedules (*n* = 11), social life (*n* = 8), and family life schedules (*n* = 5)
Kesztyüs et al. (21)	Pre-post	T: *n* = 63; C: *n* = 61	Healthy, employed	47.8 (SD = 10.5)	8–9 h (self-selected)	12 weeks	Work	11 of 61	18% of participants did not report good compatibility of TRE with work schedules
Kesztyüs et al. (22)	Pre-post	T: *n* = 40; C: *n* = 38	At least 1 component of metabolic syndrome (63% on daily medication)	49.1 (SD = 12.4)	8–9 h (self-selected)	12 weeks	Work and physical/ psychological	Work: 6/38; Hunger: 13/38	25 participants combined TRF very well or well with their daily work routine, 6 badly or very badly, and 6 were in between. Daily hunger was reported by 3, several days per week by 10, and once a week or less by 22 participants
Bjerre et al. (23)	Interviews from RCT	T: *n* = 17; C: *n* = 17	BMI ≥ 30 and eating window of ≥ 12 h and at least 1 day/week ≥ 14 h	46–68	10 h (between 6 am and 8 pm); self-selected	12 weeks	Work and social	Not reported	The largest barriers were social evening activities and societal constraints such as normal working and operating hours of establishments
Parr et al. (24)	Pre-post	T: *n* = 24; C: *n* = 19	Type-II diabetes	50 (SD = 9.0)	9 h (10 am−7 pm)	4 weeks	Family, work, social, and physical/ psychological	Not reported	Hunger and eating before 7 pm were difficult to integrate with social, work, and family commitments. Nighttime snacking due to stress or boredom
Przulj et al. (18)	Pre-post	T: *n* = 52; C: *n* = 50	Obesity with BMI ≥ 30 kg/m^2^ or >28 kg/m^2^ with comorbidities	50.1	8 h (self-selected)	12 weeks	Social	Not reported	The most common barriers were social occasions including eating out, hosting visitors, and having drinks after work
Lee et al. (25)	Pre-post	T: *n* = 10; C: *n* = 9	Overweight and sedentary	77.1	Gradual adjustment to 8 h TRE (self-selected)	4 weeks	Social and work	Not reported	Barriers included social eating events during the fasting window and changes in work schedules
Vidmar et al. (26)	RCT	T: *n* = 50; C: *n* = 45	Adolescents with BMI ≥ 95th percentile	14–18	8 h (self-selected)	12 weeks	Social, family, and work	7 of 45	Participants reported barriers including incompatibility with work or sleep schedule, conflict with social events, and difficulty explaining dietary intervention to family

### 2.2. Social events

Eight of 10 TRE trials in this analysis reported social commitments as a barrier to TRE (Table 1). In a 10-week TRE trial where each participant's eating windows were shortened by 3 h, social eating and drinking events were the most commonly reported barriers. Nine of 16 participants felt TRE was unsustainable beyond 10 weeks mainly due to an incompatible social and family life (20). Similarly, participants in a 4-week 8-h TRE trial reported that food served at social events during the fasting period was a challenge to TRE adherence (25). In another trial with an 8-h eating window ending at 5 pm, having a social life was one of three main barriers identified by participants (23). Importantly, this study did not allow a self-selected eating window, so participants were required to finish their last meal between 5 and 5:30 pm. As the average dinner time in the U.S. occurs at about 6:24 pm (27), the inability to eat later in the evening may have limited nighttime social activities.

Studies that allowed a self-selected eating window, however, did not necessarily resolve conflicts between TRE and social events: in a 12-week long trial with self-selected 8-h eating windows, participants reported dining out, having visitors, and having drinks after work as the most frequent social barriers. This study also noted that adherence to TRE during weekends decreased over time, though the reason for this was unspecified (18). Bjerre et al. (23) highlighted the importance of having adequate social support during TRE trials, citing experiences of participants whose eating windows would end in the middle of a three-course meal, or were continually offered food by those around them after their eating window had ended. At the risk of seeming impolite, participants often chose to fit into their social context by continuing to eat during their fasting window. Self-selection of an eating window, while certainly helpful for catering to variations across participants' schedules, cannot entirely insulate each individual from social influences. Eating is often a shared experience that provides important opportunities to socialize, so having the support and understanding of one's social circle may likely increase a participant's ability to follow a TRE protocol.

### 2.3. Family

Another barrier in TRE trials was the conflict between eating windows and family-related commitments. Some participants expressed that family needs came first in their household; a TRE protocol requiring an early dinner would disrupt family schedules and would therefore be impractical (24). Similarly, participants in a shorter 4-week-long TRE trial reported that eating their last meal before 7 pm was difficult to integrate with both family and work commitments (19). Implementing a TRE schedule that interferes with regular family dinnertimes would likely negatively impact long-term adherence, as family dinners in the US occur frequently in many households—an average of 4.1 times per week (28). In contrast, one trial noted that family support was critical to TRE adherence: spouses of enrolled participants adjusted their eating habits to be in synchrony with the TRE schedule (25).

### 2.4. Work

The average age of participants ranged from 29 to 77 years old (Table 1), so work schedules sometimes interfered with TRE. During a 4-week long trial, participants reported that changes in work schedules impeded TRE adherence (25). Similarly, busy workdays in which food was not able to be consumed rendered the TRE protocol to be difficult or entirely impossible (24). Other challenges included hunger at work and difficulties with integrating TRE protocol with shift work schedules (22). Despite work-related challenges, one study's survey results indicated that participants seemed to have a positive attitude overall toward their experiences with integrating work schedules with TRE: 78% of 63 participants felt TRE integrated well with their professional activities, while only 18% reported experiencing difficulties with work and TRE conflicts, and 3% considered it neither good nor difficult (21).

### 2.5. Psychological/physical factors

Of the few studies that collected participant feedback regarding specific barriers to TRE, psychological or physical factors such as stress, boredom, and hunger did not seem to significantly affect adherence (19, 22, 23). In one trial, hunger was reported as a difficulty by five out of 40 participants during the initial few weeks of the intervention period (22). Parr et al. (24) found that participants ate larger meals to avoid hunger during the fasting period. Similarly, concerns about potential hunger drove some participants to consume more food than usual during their eating window despite not feeling hungry (23). This behavior decreased as the intervention progressed, though it is unclear to what extent it negatively affected adherence. In most TRE studies, however, researchers have noted an overall decrease in calories.

### 2.6. Participants' reported strategies for overcoming barriers

Participants reported using a variety of strategies to overcome the aforementioned barriers.

#### 2.6.1. Planning ahead

Some participants prepared meals ahead of time in case they did not have enough time to cook before their eating window ended, while others carried food with them for easy access. Few participants also set alarms to remind them when to begin cooking dinner, or when to stop eating (23). The benefits of planning to increase protocol adherence are compounded by food choices, as participants who did not plan reported reaching for less healthy options to adhere to their eating window (20).

#### 2.6.2. Activities

For some, eating an earlier dinner meant creating a void in activity at night in which they felt bored. Coping methods included filling time with other usual activities (e.g., watching television, reading, and sitting at the computer), or simply going to bed earlier. In one study, some participants woke up later as a strategy to avoid early morning hunger while waiting for their eating window to begin (23). When zero-calorie beverages were permitted while fasting, participants reported that drinking water or black coffee helped to distract from hunger (18).

## 3. Potential strategies to increase adherence: American Health Association's Evidence-based strategies to enhance adherence to changes in diet and eating behaviors

Sixty-six TRE trials were examined for the following components: study design, daily eating window duration, intervention duration, method of tracking adherence, and adherence rate. We were specifically interested in the studies' method(s) of tracking adherence to TRE, which were categorized based on the American Health Association (AHA)/American College of Cardiology's (ACC) evidence-based strategies to enhance adherence to changes in diet and eating behaviors (29). These strategies have been used to encourage changes in dietary composition and intake—such as following a Mediterranean diet, or reducing alcohol and sugar intake—but have not yet been applied to TRE. Strategies include goal setting, self-monitoring, tailoring the regimen, ongoing contact, reinforcement, and social support.

### 3.1. Goal setting

A critical aspect of goal-setting in the context of TRE is setting realistic expectations at the beginning of the intervention. The reality of any dietary intervention is that some level of sacrifice and compromise needs to be made. For example, the design of TRE makes it nearly impossible for someone to be able to eat breakfast before a regular work shift (at or before 9 am) while also grabbing drinks in the evening. Similarly, participants with families may not be able to have both breakfast and dinner with their spouses and/or children on weekdays. Thus, study coordinators should work with participants to make a judgment call as to how their current lifestyle may fit in with the suggested TRE protocol, evaluate the aspects of their current eating patterns that are the most important to them, and prioritize accordingly.

### 3.2. Self-monitoring

Self-monitoring, as defined by AHA, is “systematically observing and recording one's behavior.” In the context of TRE, it refers to any form of self-reported data from participants regarding their adherence. The advantages of incorporating self-monitoring in TRE trials are two-fold: researchers can gather data on adherence rates, and participants may feel an increased sense of accountability when their daily choices are being monitored. In a 4-week TRE trial where meals were recorded on a smartphone app, Parr et al. (19) suggested that self-monitoring allowed participants to identify patterns/relationships between their eating habits and their health, particularly between dietary choices and blood glucose, which increased overall feelings of self-awareness and accountability.

A total of 21 studies utilized daily self-monitoring, in which participants recorded either (1) all food intake on a phone app, or (2) the timing and/or content of their first and last calorie in a written diary or electronically (Table 2). As with any form of self-reporting, a drawback of self-monitoring is the possibility of inaccurately reported data and increased user burden. Using a smartphone application (e.g., Easy Diet Diary or myCircadianClock) that automatically generates time stamps with food entries can increase the accuracy of eating window data. myCircadianClock, the only TRE research app created by a research team, is functional on both Android and iPhones (38) and allows participants to record food intake with a brief note and a picture. Study coordinators are also able to remotely check participants' eating histories and daily progress on protocol adherence. However, meals eaten can still be omitted by the participant—intentionally or not—which highlights the need for self-monitoring to be used as part of a multi-faceted approach to managing adherence.

**Table 2:** Adherence rates and methods used to encourage adherence.

**References**	**Study design**	**Eating window**	**Intervention duration**	**Method of tracking and encouraging adherence**	**Evidence-based method to increase adherence**	**Adherence to TRE**
Chow et al. (30)	RCT	8 h (self-selected start time)	12 weeks	Daily food logging in myCircadianClock app	Self-monitoring, ongoing contact, and reinforcement	66%
Przulj et al. (18)	Pre-post	8 h (self-selected start time)	12 weeks	Clinic visits at 1, 6, and 12 weeks. Phone calls at weeks 2, 3, 4, and 5. Participants were also given a diary card to log their daily eating window and keep track of their TRE adherence/hunger ratings	Ongoing contact and self-monitoring	71%
Parr et al. (19)	Pre-post	9 h (10 am−7 pm)	4 weeks	Self-monitoring smartphone app (EasyDietDiary) to record all food entries. Photos of each food/beverage were taken using their phone	Self-monitoring	72%
Kesztyüs et al. (21)	Pre-post	8–9 h (self-selected start time)	12 weeks	The daily food journal of first and last calorie	Self-monitoring	72%
Kesztyüs et al. (16)	Secondary analysis of 2 pre-post studies	8–9 h (self-selected start time)	12 weeks	The daily food journal of first and last calories	Self-monitoring	77%
Gabel et al. (31)	Single arm with matched historical control with weight loss trial	8 h (10 am−6 pm)	12 weeks	Daily adherence log (first and last calorie). And 7-day food journal during baseline and week 12 of intervention	Self-monitoring	80%
Gabel et al. (11)	Pre-post	8 h (10 am−6 pm)	12 weeks	Daily adherence log (first and last calorie) and 7-day food journal during baseline and week 12 of intervention	Self-monitoring	80%
Anton et al. (32)	Pre-post	8 h (12 h for the first week)	4 weeks	The daily food journal of first and last calories. Contacted *via* phone at the end of weeks 1, 2, and 3 to review protocol and discuss any adverse events	Self-monitoring and ongoing contact	84%
Martens et al. (33)	RCT	8 h (start time between 10–11 am)	6 weeks	A daily electronic survey administered *via* email; is sent at 7 pm daily. Participants reported their first and last eating events	Self-monitoring	84%
Kesztyüs et al. (22)	Pre-post	8–9 h (self-selected start time)	12 weeks	The daily food journal of first and last calories. Telephone call 2–3 weeks after fasting initiation to discuss coping with fasting, whether they noticed changes, how they felt about their health, and if any problems arose	Self-monitoring and ongoing contact	86%
Cienfuegos et al. (1)	RCT (3 arm)	4-h (3–7 pm), 6-h (1–7 pm)	8 weeks	Daily adherence log (first and last calorie) and weekly review with the study coordinator, who would emphasize the importance of eating within the window at the end of each meeting	Self-monitoring, ongoing contact, and reinforcement	89%
Wilkinson et al. (6)	Pre-post	10 h (self-selected start time)	12 weeks	Daily food logging in myCircadianClock app. Participants received educational tips and reminders *via* in-app push notifications. Participants who were non-adherent or not logging sufficiently were contacted	Self-monitoring, ongoing contact, and reinforcement	94%
Brady et al. (34)	RCT	8 h (self-selected start time)	8 weeks	Daily food log of eating windows. Full food diaries at baseline, 4 weeks, and 8 weeks	Self-monitoring	95%
Lowe et al. (35)	RCT	8 h (12 pm−8 pm)	12 weeks	A self-monitoring smartphone app that asked participants if they were compliant with TRE every day	Self-monitoring	84%
Prasad et al. (8)	Pre-post	10 h (self-selected start time)	90 days (12.8 weeks)	Daily food logging in myCircadianClock app. Study coordinators monitored participants and sent reminders to log in/use the app *via* text or in-app push notifications	Self-monitoring and ongoing contact	47%
Vidmar et al. (26)	RCT	8 h (self-selected start time)	12 weeks	Self-recorded start/stop times of daily food intake and reported these times to study staff each week. 24 h dietary recall was conducted at three-time points during the study	Self-monitoring and ongoing contact	74%
Zhang et al. (36)	RCT	6 h (eTRE: 7 am−1 pm, lTRE: 12 pm−6 pm)	10 weeks	Daily adherence log used to record start/stop times of eating	Self-monitoring	eTRE: 89% lots: 78%
Kleckner et al. (37)	RCT	10 h (self-selected start time)	2 weeks	Daily food diary of first and last calories. 24 h dietary recall was conducted at Baseline and day 14	Self-monitoring, Ongoing contact	90%
Manoogian et al. (38)	RCT	10 h (self-selected start time)	12 weeks	Daily food logging in myCircadianClock app. Participants received educational information and reminders *via* in-app push notifications to encourage engagement and adherence. Study coordinators monitored participants 2–3 times/week and contacted participants who were non-adherent or not logging sufficiently	Self-monitoring, ongoing contact, and reinforcement	71%
Haganes et al. (39)	RCT	<10 h	7 weeks	Participants reported their daily eating window. All participants were contacted *via* phone/e-mail by study investigators every week to provide support and encouragement	Self-monitoring and ongoing contact	86%
Jamshed et al. (40)	RCT	8 h (7 am−3 pm)	14 weeks	Participants reported their daily eating window through surveys administered *via* REDCap software. Eating within their assigned window within 30 mins was considered adherent	Self-monitoring	86%

The adherence rate refers to the percentage of days during intervention when participants were compliant with their TRE protocol. Studies that were shorter than 4 weeks, did not include some form of daily monitoring of compliance or did not report their participants' protocol adherence rates were not included. Studies' methods of tracking and encouraging adherence included self-monitoring (SM), ongoing contact (OC), and reinforcement (R).

It is difficult to examine how incorporating self-monitoring may have affected adherence to TRE in prior studies, as many studies did not monitor or report on rates of adherence. This also is a likely contributor to differences in outcomes between studies. Nonetheless, it is clear from other behavioral interventions, such as caloric restriction, that incorporating daily self-monitoring is necessary to track participant adherence throughout a study. A caloric restriction requires calorie tracking at baseline and throughout the intervention, and thus the timing of dietary intake must be tracked throughout TRE interventions as well.

### 3.3. Tailoring the regimen

One method of facilitating a smoother implementation of TRE is allowing each individual to self-select an eating window. As the timing of caloric consumption is the focus of time-restricted eating, it will benefit participants to be judicious in selecting an eating window that fits their needs. Potential considerations include sleep/wake patterns, work schedules, and care responsibilities. Tailoring the regimen can also include making minor adjustments to an individual's TRE protocol throughout the study. For example, if a participant is experiencing intense caffeine withdrawal in the mornings, they might be allowed to shift their eating window earlier by 1 h for the rest of the study. Or, if the family dinner is at 6:30 pm, then they could set an eating window that encompasses this event.

### 3.4. Ongoing contact

TRE interventions can last several months; therefore, contacting participants throughout is key. Ongoing contact can vary in terms of content, frequency, and method of contact. Past studies contacted participants to check in to assess any potential side effects, send reminders for protocol adherence, or answer questions. It may be especially important to contact participants during the first few weeks of intervention to ensure clear comprehension of the protocol (e.g., calorie vs. no-calorie foods that break a fast) (25). Getting through holidays or vacations can also require extra support, both during and after. Ongoing contact can also help to address socio-environmental barriers by identifying challenging aspects of participants' environments and either implementing behavioral change strategies and/or modifying the TRE protocol accordingly.

The frequency of contact can depend on numerous factors including the length of intervention, participant compliance, and participant preference. Participants exhibiting difficulties with protocol compliance may need to be contacted regularly for reminders and encouragement, while busier participants may not wish to be contacted so frequently.

The third consideration is the method of contact. Phone calls and emails can provide in-depth forms of exchange, which can be used in combination with text messages and push notifications *via* smartphone applications that provide a brief method of contact for non-urgent matters. Preference for the method of contact may also differ for each participant and should be accommodated when possible.

### 3.5. Reinforcement

Reinforcement refers to any positive feedback given to participants on their progress. In the context of TRE, positive feedback can be given to participants to reinforce good behaviors such as consistently recording their meals and adhering to their eating window. In longer trials, sending reinforcement can be a great way to acknowledge participants for their efforts while encouraging them to persist. Similarly, reinforcement can also be used to encourage compliance with TRE while emphasizing any positive elements of the participant's participation to prevent negative feelings (e.g., guilt or shame) toward their progress. Ongoing contact and reinforcement frequently overlap as study coordinators often contact participants *via* phone, email, or push notifications to send encouragement and reminders for protocol adherence.

### 3.6. Social support

Finding support in work, social, and home settings is essential for encouraging adherence to TRE. Support can mean finding individuals that share similar goals—following a similar TRE schedule, for example—or simply finding individuals that support the participant's change in eating habits. Receiving support from family and social circles can mean fewer temptations to break a fast and encountering less friction in response to altered behavior (e.g., not eating the last course at a dinner party, avoiding alcohol at a social event, leaving an event earlier, etc.). Similar to having healthy food choices available at home and removing items of temptation, having everyone in the household on the same eating schedule is an important environmental factor for adopting and maintaining TRE.

## 4. Suggestions for future studies

### 4.1. Protocol flexibility

To address these barriers in future trials, two of the studies suggested possible alterations in protocol to enhance the flexibility, and therefore feasibility, of TRE. As most social events take place at night, particularly on weekends, allowing occasional off days as part of the TRE protocol could potentially enhance its feasibility as a long-term intervention. Specifically, Przulj et al. (18) and Parr et al. (24) suggested implementing TRE for 5 out of 7 days per week to encourage long-term adherence. However, it should be noted that without official off days, participant adherence is typically 5/7 days (Table 2). Thus, if only 5 days of TRE adherence are required, adherence may decrease to 3–4 days instead. Although most trials that report adherence to TRE as 5/7 days still conclude the efficacy of the intervention, the lack of consistency in establishing a circadian routine should be avoided as it could contribute to circadian disruption (41). Overall, further research is required to better understand how the frequency of eating outside of the eating window impacts the positive health outcomes associated with TRE. Another potential accommodation would be to allow participants to eat outside their designated window for special occasions or social events up to once or twice a week as necessary.

### 4.2. Eating window customization

In light of challenges faced by participants in prior TRE trials, future trials may wish to tailor their protocol to address barriers from multiple angles. Potential considerations include the participants' family/home life (Do they have family meals often?), social life (How frequently do they eat out? When do they usually socialize?), and work schedule (Are they currently employed—and if so, is their schedule able to accommodate TRE?). Additionally, it is important to consider how culture and religion may play a role in the timing of food intake. It is worth noting that most TRE trials have been conducted in limited cultural environments and thus future research would benefit from exploring the full implications of cultural and religious influences. While a singular eating window length that is optimal for health has not yet been identified, previous trials have shown that a longer eating window of 12 h does not yield the same health benefits (42), while a shorter eating window of 4 h doesn't show additional benefits compared to 6-h TRE (1). Given the current body of literature, eating windows ranging from 6 to 10 h seems to be ideal as they yield benefits while allowing participants to eat within a reasonable time frame (5, 6, 43). Moreover, some studies have shown that an earlier window may be more beneficial than an eating window that ends later in the day (9, 44), yet many benefits are still seen from TRE studies with a later eating window (45). The importance of aligning food intake with the active phase of an individual is well-studied and should be taken into consideration when selecting an eating window.

### 4.3. Easing implementation

Some potential protocol alterations to address barriers that have not been extensively studied include implementing (1) scheduled off-days from TRE, and (2) a gradual reduction in the eating window to reach targeted fasting hours. Participant self-monitoring can be a great way to track dietary habits and increase self-accountability, while other methods targeting behavior change can reinforce positive progress while proactively preventing deviations from the protocol. Being proactive with these methods rather than waiting for issues to arise before contacting participants may also encourage adherence.

### 4.4. Education

In clinical trials aimed at exploring TRE for a particular illness/disease, it may also be useful to evaluate participants' perceptions of the severity and risk of leaving the illness/disease untreated. The Health Belief Model (HBM) suggests that a person's likelihood of changing their behavior can be predicted by their perceived severity of illness along with perceptions of the effectiveness of the proposed health intervention, so leveraging aspects of belief (i.e., perceptions of severity, risk, and benefits) may be worthwhile (46).

### 4.5. Daily monitoring

Daily monitoring of dietary intake is a key component of assessing adherence and should thus be a core component of all TRE trials.

### 4.6. Behavioral science insights

Behavioral science indicates that ongoing support and interaction are essential components of ensuring adherence to any lifestyle intervention, including TRE. Thus, future research should emphasize the importance of continuous assessment and support to help participants maintain behavioral changes.

## 5. Limitations

Studies that collected participant feedback on barriers often had small sample sizes, and there is currently no standardized method of collecting feedback on TRE barriers. As a result, there is wide variation in the type of data collected and how it is reported, making it difficult to identify salient patterns across many trials. Most studies that collected data on TRE barriers did not report the exact number of participants experiencing each barrier. As TRE is a relatively new dietary intervention, more data is needed to conclude (a) overall adherence rates in TRE trials, (b) the types of barriers participants face and the frequencies at which they occur, and (c) the effectiveness of behavioral change strategies outlined in this review.

## 6. Conclusion

TRE has shown promise in clinical trials as a simple yet effective dietary intervention. As a straightforward protocol that solely focuses on the timing of food intake, participants are not required to restrict or change any aspect of their regular diet. Nonetheless, the long-term feasibility of this protocol is less understood, which merits a closer examination of barriers faced by participants during trials. To increase protocol adherence—and therefore feasibility—of TRE, future trials may consider exploring a combination of health behavioral change strategies, tracking adherence closely, and soliciting participant feedback on TRE barriers.

## Author contributions

MO'N, KL, and NG performed the literature review and compiled data. NG conceptualized the paper and completed the initial literature review and contributed to the writing. MO'N wrote the draft of the manuscript. MO'N, KL, EM, and SP edited the manuscript. EM and SP provided feedback and mentorship at all stages. All authors contributed to the article and approved the submitted version.

## References

[1] Cienfuegos S Gabel K Kalam F Ezpeleta M Wiseman E Pavlou V. Effects of 4- and 6-h time-restricted feeding on weight and cardiometabolic health: a randomized controlled trial in adults with obesity. Cell Metab. (2020) 32:366–78e3. 10.1016/j.cmet.2020.06.018 32673591 PMC9407646

[2] Chaix A Manoogian ENC Melkani GC Panda S. Time-restricted eating to prevent and manage chronic metabolic diseases. Annu Rev Nutr. (2019) 39:291–315. 10.1146/annurev-nutr-082018-124320 31180809 PMC6703924

[3] Moon S Kang J Kim SH Chung HS Kim YJ Yu JM. Beneficial effects of time-restricted eating on metabolic diseases: a systemic review and meta-analysis. Nutrients. (2020) 12:e1267. 10.3390/nu12051267 32365676 PMC7284632

[4] Chantranupong L Wolfson RL Sabatini DM. Nutrient-sensing mechanisms across evolution. Cell. (2015) 161:67–83. 10.1016/j.cell.2015.02.041 25815986 PMC4384161

[5] Zhao L Hutchison AT Liu B Yates CL Teong XT Wittert GA. Time-restricted eating improves glycemic control and dampens energy- consuming pathways in human adipose tissue. Nutrition. (2022) 96:111583. 10.1016/j.nut.2021.111583 35150947

[6] Wilkinson MJ Manoogian ENC Zadourian A Lo H Fakhouri S Shoghi A. Ten-hour time-restricted eating reduces weight, blood pressure, and atherogenic lipids in patients with metabolic syndrome when to eat: the importance of eating patterns in health and disease. Cell Metab. (2020) 31:92–104 e5. 10.1016/j.cmet.2019.11.004 31813824 PMC6953486

[7] Hutchison AT Regmi P Manoogian ENC Fleischer JG Wittert GA Panda S. Time-restricted feeding improves glucose tolerance in men at risk for type 2 diabetes: a randomized crossover trial. Obesity (Silver Spring). (2019) 27:724–32. 10.1002/oby.22449 31002478

[8] Prasad M Fine K Gee A Nair N Popp CJ Cheng B. A smartphone intervention to promote time restricted eating reduces body weight and blood pressure in adults with overweight and obesity: a pilot study. Nutrients. (2021) 13:2148. 10.3390/nu13072148 34201442 PMC8308240

[9] Sutton EF Beyl R Early KS Cefalu WT Ravussin E Peterson CM. Early time-restricted feeding improves insulin sensitivity, blood pressure, and oxidative stress even without weight loss in men with prediabetes. Cell Metab. (2018) 27:1212–21.e3. 10.1016/j.cmet.2018.04.010 29754952 PMC5990470

[10] Moro T Tinsley G Bianco A Marcolin G Pacelli QF Battaglia G. Effects of eight weeks of time-restricted feeding (16/8) on basal metabolism, maximal strength, body composition, inflammation, and cardiovascular risk factors in resistance-trained males. J Transl Med. (2016) 14:290. 10.1186/s12967-016-1044-0 27737674 PMC5064803

[11] Gabel K Marcell J Cares K Kalam F Cienfuegos S Ezpeleta M. Effect of time restricted feeding on the gut microbiome in adults with obesity: a pilot study. Nutr Health. (2020) 26:79–85. 10.1177/0260106020910907 32228124

[12] Tinsley GM Moore ML Graybeal AJ Paoli A Kim Y Gonzales JU. Time-restricted feeding plus resistance training in active females: a randomized trial. Am J Clin Nutr. (2019) 110:628–40. 10.1093/ajcn/nqz126 31268131 PMC6735806

[13] Domaszewski P Konieczny M Pakosz P Baczkowicz D Sadowska-Krepa E. Effect of a six-week intermittent fasting intervention program on the composition of the human body in women over 60 years of age. Int J Environ Res Public Health. (2020) 17:4138. 10.3390/ijerph17114138 32531956 PMC7312819

[14] Li C Xing C Zhang J Zhao H Shi W He B. Eight-hour time-restricted feeding improves endocrine and metabolic profiles in women with anovulatory polycystic ovary syndrome. J Transl Med. (2021) 19:148. 10.1186/s12967-021-02817-2 33849562 PMC8045367

[15] Gill S Panda S. A smartphone app reveals erratic diurnal eating patterns in humans that can be modulated for health benefits. Cell Metab. (2015) 22:789–98. 10.1016/j.cmet.2015.09.005 26411343 PMC4635036

[16] Kesztyüs D Fuchs M Cermak P Kesztyüs T. Associations of time-restricted eating with health-related quality of life and sleep in adults: a secondary analysis of two pre-post pilot studies. BMC Nutr. (2020) 6:76. 10.1186/s40795-020-00402-2 33327959 PMC7745395

[17] Kim H Jang BJ Jung AR Kim J Ju HJ Kim YI. The impact of time-restricted diet on sleep and metabolism in obese volunteers. Medicina (Kaunas). (2020) 56:540. 10.3390/medicina56100540 33066554 PMC7602198

[18] Przulj D Ladmore D Smith KM Phillips-Waller A Hajek P. Time restricted eating as a weight loss intervention in adults with obesity. PLoS ONE. (2021) 16:e0246186. 10.1371/journal.pone.0246186 33508009 PMC7842957

[19] Parr EB Devlin BL Lim KHC Moresi LNZ Geils C Brennan L. Time-restricted eating as a nutrition strategy for individuals with type 2 diabetes: a feasibility study. Nutrients. (2020) 12:e3228. 10.3390/nu12113228 33105701 PMC7690416

[20] Antoni R Robertson TM Robertson MD Johnston JD. A pilot feasibility study exploring the effects of a moderate time-restricted feeding intervention on energy intake, adiposity and metabolic physiology in free-living human subjects. J Nutr Sci. (2018) 7:E22. 10.1017/jns.2018.13

[21] Kesztyüs D Vorwieger E Schönsteiner D Gulich M Kesztyüs T. Applicability of time-restricted eating for the prevention of lifestyle-dependent diseases in a working population: results of a pilot study in a pre-post design. Ger Med Sci. (2021) 19:Doc04. 10.21203/rs.2.20835/v1 33911996 PMC8051591

[22] Kesztyüs D Cermak P Gulich M Kesztyüs T. Adherence to time-restricted feeding and impact on abdominal obesity in primary care patients: results of a pilot study in a pre-post design. Nutrients. (2019) 11:2854. 10.3390/nu11122854 31766465 PMC6950236

[23] Bjerre N Holm L Quist JS Færch K Hempler NF. Watching, keeping and squeezing time to lose weight: Implications of time-restricted eating in daily life. Appetite. (2021) 161:105138. 10.1016/j.appet.2021.105138 33524440

[24] Parr EB Devlin BL Radford BE Hawley JA. A delayed morning and earlier evening time-restricted feeding protocol for improving glycemic control and dietary adherence in men with overweight/obesity: a randomized controlled trial. Nutrients. (2020) 12:505. 10.3390/nu12020505 32079327 PMC7071240

[25] Lee SA Sypniewski C Bensadon BA McLaren C Donahoo WT Sibille KT. Determinants of adherence in time-restricted feeding in older adults: lessons from a pilot study. Nutrients. (2020) 12:874. 10.3390/nu12030874 32213965 PMC7146127

[26] Vidmar AP Naguib M Raymond JK Salvy SJ Hegedus E Wee CP. Time-limited eating and continuous glucose monitoring in adolescents with obesity: A pilot study. Nutrients. (2021) 13:3697. 10.3390/nu13113697 34835953 PMC8624400

[27] Kant AK. Eating patterns of US adults: meals, snacks, and time of eating. Physiol Behav. (2018) 193(Pt B):270–8. 10.1016/j.physbeh.2018.03.022 29574043

[28] Larson N MacLehose R Fulkerson JA Berge JM Story M Neumark-Sztainer D. Eating breakfast and dinner together as a family: associations with sociodemographic characteristics and implications for diet quality and weight status. J Acad Nutr Diet. (2013) 113:1601–9. 10.1016/j.jand.2013.08.011 24139290 PMC3833880

[29] Van Horn L Carson JA Appel LJ Burke LE Economos C Karmally W. Recommended dietary pattern to achieve adherence to the American Heart Association/American College of Cardiology (AHA/ACC) Guidelines: A Scientific Statement From the American Heart Association. Circulation. (2016) 134:e505–29. 10.1161/CIR.0000000000000462 27789558

[30] Chow LS Manoogian ENC Alvear A Fleischer JG Thor H Dietsche K. Time-restricted eating effects on body composition and metabolic measures in humans who are overweight: A feasibility study. Obesity (Silver Spring). (2020) 28:860–9. 10.1002/oby.22756 32270927 PMC7180107

[31] Gabel K Hoddy KK Haggerty N Song J Kroeger CM Trepanowski JF. Effects of 8-hour time restricted feeding on body weight and metabolic disease risk factors in obese adults: A pilot study. Nutr Healthy Aging. (2018) 4:345–53. 10.3233/NHA-170036 29951594 PMC6004924

[32] Anton SD Lee SA Donahoo WT McLaren C Manini T Leeuwenburgh C. The effects of time restricted feeding on overweight, older adults: A pilot study. Nutrients. (2019) 11:1500. 10.3390/nu11071500 31262054 PMC6682944

[33] Martens CR Rossman MJ Mazzo MR Jankowski LR Nagy EE Denman BA. Short-term time-restricted feeding is safe and feasible in non-obese healthy midlife and older adults. Geroscience. (2020) 42:667–86. 10.1007/s11357-020-00156-6 31975053 PMC7206473

[34] Brady AJ Langton HM Mulligan M Egan B. Effects of 8 wk of 16:8 Time-restricted eating in male middle- and long-distance runners. Med Sci Sports Exerc. (2021) 53:633–42. 10.1249/MSS.0000000000002488 32796255

[35] Lowe DA Wu N Rohdin-Bibby L Moore AH Kelly N Liu YE. Effects of time-restricted eating on weight loss and other metabolic parameters in women and men with overweight and obesity: The TREAT randomized clinical trial. JAMA Intern Med. (2020) 180:1–9. 10.1001/jamainternmed.2020.4153 32986097 PMC7522780

[36] Zhang LM Liu Z Wang JQ Li RQ Ren JY Gao X. Randomized controlled trial for time-restricted eating in overweight and obese young adults. iScience. (2022) 25:104870. 10.1016/j.isci.2022.104870 36034217 PMC9400087

[37] Kleckner AS Altman BJ Reschke JE Kleckner IR Culakova E Dunne RF. Time-restricted eating to address cancer-related fatigue among cancer survivors: A single-arm pilot study. J Integr Oncol. (2022) 11:367. 36131848 PMC9489052

[38] Manoogian ENC Wei-Shatzel J Panda S. Assessing temporal eating pattern in free living humans through the myCircadianClock app. Int J Obes. (2022) 46:696–706. 10.1038/s41366-021-01038-3 34997205 PMC9678076

[39] Haganes KL Silva CP Eyjólfsdóttir SK Steen S Grindberg M Lydersen S. Time-restricted eating and exercise training improve HbA1c and body composition in women with overweight/obesity: A randomized controlled trial. Cell Metab. (2022) 34:1457–71.e4. 10.1016/j.cmet.2022.09.003 36198292

[40] Jamshed H Steger FL Bryan DR Richman JS Warriner AH Hanick CJ. Effectiveness of early time-restricted eating for weight loss, fat loss, and cardiometabolic health in adults with obesity: A randomized clinical trial. JAMA Intern Med. (2022) 182:953–62. 35939311 10.1001/jamainternmed.2022.3050 PMC9361187

[41] Fishbein AB Knutson KL Zee PC. Circadian disruption and human health. J Clin Invest. (2021) 131:e148286. 10.1172/JCI148286 34596053 PMC8483747

[42] Phillips NE Mareschal J Schwab N Manoogian ENC Borloz S Ostinelli G. The effects of time-restricted eating versus standard dietary advice on weight, metabolic health and the consumption of processed food: a pragmatic randomised controlled trial in community-based adults. Nutrients. (2021) 13:1042. 10.3390/nu13031042 33807102 PMC8004978

[43] Crose A Alvear A Singroy S Wang Q Manoogian E Panda S. Time-restricted eating improves quality of life measures in overweight humans. Nutrients. (2021) 13:1430. 10.3390/nu13051430 33922683 PMC8146708

[44] Regmi P Heilbronn LK. Time-restricted eating: benefits, mechanisms, and challenges in translation. iScience. (2020) 23:101161. 10.1016/j.isci.2020.101161 32480126 PMC7262456

[45] Manoogian EN Chow LS Taub PR Laferrère B Panda S. Time-restricted eating for the prevention and management of metabolic diseases. Endocr Rev. (2021) 43:405–36. 10.1210/endrev/bnab027 34550357 PMC8905332

[46] Rosenstock I. The health belief model and preventive health behavior. Health Educ Monogr. (1974) 2:354–86. 10.1177/109019817400200405

